# Considering strategies for SNP selection in genetic and polygenic risk scores

**DOI:** 10.3389/fgene.2022.900595

**Published:** 2022-10-25

**Authors:** Julien St.-Pierre, Xinyi Zhang, Tianyuan Lu, Lai Jiang, Xavier Loffree, Linbo Wang, Sahir Bhatnagar, Celia M. T. Greenwood, CANSSI team on Improving Robust High-Dimensional Causal Inference and Prediction Modelling

**Affiliations:** ^1^ Department of Epidemiology, Biostatistics and Occupational Health, McGill University, Montréal, QC, Canada; ^2^ Department of Statistical Sciences, University of Toronto, Toronto, ON, Canada; ^3^ Lady Davis Institute for Medical Research, Jewish General Hospital, Montréal, QC, Canada; ^4^ Quantitative Life Sciences, McGill University, Montréal, QC, Canada; ^5^ Department of Statistics and Actuarial Sciences, University of Waterloo, Waterloo, ON, Canada; ^6^ Gerald Bronfman Department of Oncology, McGill University, Montréal, QC, Canada

**Keywords:** polygenic risk scores, measurement error, instrumental variable methods, mendelian randomization, regularized models, high-dimensional data, feature selection

## Abstract

Genetic risk scores (GRS) and polygenic risk scores (PRS) are weighted sums of, respectively, several or many genetic variant indicator variables. Although they are being increasingly proposed for clinical use, the best ways to construct them are still actively debated. In this commentary, we present several case studies illustrating practical challenges associated with building or attempting to improve score performance when there is expected to be heterogeneity of disease risk between cohorts or between subgroups of individuals. Specifically, we contrast performance associated with several ways of selecting single nucleotide polymorphisms (SNPs) for inclusion in these scores. By considering GRS and PRS as predictors that are measured with error, insights into their strengths and weaknesses may be obtained, and SNP selection approaches play an important role in defining such errors.

## 1 Introduction

Genetic risk scores (GRS) and polygenic risk scores (PRS) are increasingly used as predictors of disease risk (Khera et al., 2018), and active discussions are ongoing on how to incorporate them effectively into health care (Inouye et al., 2018; Forgetta et al., 2020; Lu et al., 2021a). These scores can display variable abilities to accurately estimate disease risks in different contexts. For example, in Howe et al. (2020), a coronary artery disease PRS was shown to predict incident coronary artery disease (CAD) events more accurately amongst individuals with no prevalent CAD history than those with prevalent CAD; analogous examples of differential discrimination have been shown by diabetes status (Udler et al., 2019) and atherosclerotic heart disease (Aragam and Natarajan, 2020). A compelling rationale for differential score performance can be made for males versus females (Roberts et al., 2020; Manikpurage et al., 2021), since sex-specific disease incidence and regulation is the norm rather than the exception (Ober et al., 2008). Furthermore, not only are there loci whose effects vary across subgroups, but loci exist that are associated with increased prediction variability in phenotypes (Wang et al., 2019; Lu et al., 2022). Without a good understanding of the contexts where genetic or polygenic risk scores perform well, it is more challenging to argue for their clinical use.

A genetic score or polygenic score is a weighted sum of allele indicators at each element of a set of single nucleotide polymorphisms (SNPs). The distinction between GRS and PRS is loosely a function of how many SNPs are in the set, with ‘poly’genic risk scores often containing hundreds of variants, and GRS including only a few. The theoretical performance of PRS as a function of sample size, heritability, and the distribution of the true effect sizes has been expertly discussed by Dudbridge (2013) and Chatterjee et al. (2013), among others.

Commonly, SNP set inclusion is initially defined by a *p*-value filter, and then refined through one or more filtering strategies to include only variants that contribute non-redundant information. Selection of SNPs for inclusion in a GRS or PRS can be considered to be the most challenging aspect of their construction. One commonly-used approach for SNP selection is clumping and thresholding (C + T) (also termed pruning and filtering), where SNPs are selected on the basis of their individual statistical significance and low linkage disequilibrium patterns with other nearby SNPs. However, many more sophisticated methods have been developed for SNP selection after GWAS, to improve accuracy of disease risks estimates. Among the methods proposed, some attempt to increase the likelihood that the selected SNPs are truly causal. For example, fine mapping after a Genome-Wide Association Study (GWAS) predicts the most likely causal SNP(s) at a locus (e.g., (Kichaev et al., 2014; Chen et al., 2015; Benner et al., 2016; Wang et al., 2020; Zhang et al., 2021; Forgetta et al., 2022)). Even if the SNPs retained after such fine mapping algorithms are not truly causal, they are likely to be strongly correlated with the causal SNPs. Other strategies for improving SNP selection include improved characterizations of genetic architecture (Ni et al., 2021), and suggestions for keeping SNPs with relevant functional annotations (Udler et al., 2019; Amariuta et al., 2020). Score performance–i.e., accurate prediction of risks–using these more nuanced methods is usually better than simply using C + T. Shrinkage of the estimated coefficients has also been proposed with the goal of minimizing winner’s curse bias from the univariate GWAS (Zollner and Pritchard, 2007). Alternatively, rather than performing score construction based on results from GWAS, PRS can be directly estimated from the linear predictor obtained after fitting a high dimensional regression model, such as the LASSO or other penalized regression models (Lello et al., 2018; Forgetta et al., 2020; Lu et al., 2021a; Lu et al., 2021b). These approaches directly estimate independent contributions from correlated sets of SNPs without requiring explicit clumping or fine mapping, but implementation requires very large sample sizes and computational capacity.

Recently, the potential uses of GRS have gone beyond prediction of clinically relevant outcomes. Genetic scores are also being considered as tools for inferring causal relationships in the context of Mendelian randomization (MR) studies (Palmer et al., 2012; Burgess and Thompson, 2013; Davies et al., 2018). In an observational study, MR can leverage genetic variants as instruments to estimate the evidence for a causal relationship between a risk factor and an outcome (Burgess et al., 2015). Conceptually, a GRS combines the power of multiple SNPs to construct a stronger instrument, thus improving statistical power and avoiding bias in the causal effect estimate due to weak instruments (Davies et al., 2015). When all SNPs in the score satisfy the assumptions required for MR, the GRS will also be a valid instrument. However, valid inference may still be obtained even when some SNPs are invalid (e.g., Bowden et al., 2016; Guo et al., 2018; Ye et al., 2021). A brief discussion of these methods is deferred to Section 3.3.

One elemental example of variation in GRS and PRS performance is the differential performance across ancestral populations (Martin et al., 2017). These differences are partially a consequence of genetic architecture differences, i.e., different causal variants, linkage disequilibrium patterns, and allele frequencies, but they are also due to the use of European-centric genotyping panels and imputation algorithms. However, in this commentary, we will not specifically address ancestry differences in score performance, since it is a topic that would require its own manuscript to do it justice.

Instead, our goal here is, through the use of a few case studies, to examine variability in genetic score performance as a function of SNP selection and context. First we briefly describe three sets of data. In the Methods section, we briefly introduce a few different approaches for SNP selection. Then in Results, we illustrate the performance of these method choices through illustrative analyses of three datasets. Finally we discuss how SNP selection influences performance in different datasets and contexts.

## 2 Datasets

### 2.1 Temporomandibular disorder

Temporomandibular disorder (TMD) is a painful disease of the jaw. We used data on independent subjects from four cohorts containing TMD information: the Orofacial Pain: Prospective Evaluation and Risk Assessment (OPPERA) study; the Sao Paulo, Brazil, TMD case-control (SPB) study; the OPPERA II Chronic TMD Replication case-control study, and the Complex Persistent Pain Conditions (CPPC): Unique and Shared Pathways of Vulnerability study. Significant associations between TMD and three distinct loci have been previously reported in combined or sex-segregated analyses on the OPPERA cohort (Smith et al., 2018). Sample sizes and country of recruitment for the four studies are shown in Table 1, and further details on study design, recruitment, subject characteristics, and phenotyping for each study are provided in the Supplementary Materials of Smith et al. (2018) (available at http://links.lww.com/PAIN/A688).

**TABLE 1:** Demographic data for the four cohorts on Temporomandibular Disorder.

	Study name				
	Orofacial pain:prospective evaluation and risk assessment	Sao Paulo, Brazil, TMD case-control	OPPERA-II chronic TMD replication	Complex persistent PAIN conditions	Total
Acronym	OPPERA	SPB	OPPERA2	PPG	
Country	United States	Brazil	United States	United States	
N (% female)	3030 (64.6)	436 (100.0)	1342 (66.0)	390 (84.4)	5196 (69.4)
Cases (%)	999 (33.0)	144 (33.0)	444 (33.0)	164 (42.0)	1751 (33.7)
Ancestry (% white)	61	100	79	68	69

#### 2.1.1 Unrelated individuals

Before combining data across the four cohorts, we estimated the relationships between all pairs of individuals. For each pair of related subjects up to 1^st^ degree, defined such that the kinship coefficient >0.177, we removed one individual with the lowest call rate, resulting in 71 individuals being dropped. Also, we removed 199 subjects from the SPB study who were classified as having TMD without any pain.

#### 2.1.2 Quality control and pruning

Before merging the raw genotyped data (before imputation) for the four cohorts, we filtered for minor allele frequency greater than 1% and SNP call rate greater than 95%, using PLINK 1.9 (Chang et al., 2015). Then, we merged the four cohorts together into one dataset, and filtered once again for SNP call rate greater than 95%, to retain only SNPs that were present in all cohorts. This led to a total of 67,930 variants. We pruned the samples using the “indep-pairwise” option in PLINK 1.9 such that all SNPs within a window size of 100 had pairwise *r* ^2^ < 0.2. As recommended by Price et al. (2008), we also removed SNPs from a list of predetermined long-range LD regions. After pruning, 49,750 genotyped variants remained, and all of these were used to calculate principal components (PCs) of ancestry.

#### 2.1.3 Imputation of genetic data

We used the imputed data described in Smith et al. (2018). Genotypes were imputed to the 1000 Genomes Project phase 3 reference panel using the software packages SHAPEIT (Delaneau et al., 2011) for prephasing and IMPUTE2 (Howie et al., 2009). For each cohort independently, we assessed imputation quality taking into account the number of minor alleles as well as the information score such that a SNP with rare MAF must pass a higher quality information threshold for inclusion: all imputed markers satisfying the following inequality were retained in the analysis

$$2\times MAF\times \left(1-MAF\right)\times INFO\geq 0.05.$$

After merging all four cohorts, we filtered for HWE separately in cases and controls, using a more strict threshold among cases to avoid discarding disease-associated SNPs that are possibly under selection Marees et al. (2018) ( $<10^{-6}$ in controls, $<10^{-11}$ in cases). We also filtered, again, using a SNP call rate greater than 95% on the combined dataset to retain imputed variants present in all cohorts. The final merged dataset thus included a total of 4.8*M* imputed SNPs.

### 2.2 United Kingdom biobank

We followed Forgetta et al. (2022) when using data from the UK Biobank (ukbiobank.org (Bycroft et al., 2018)). We extracted 11 *Ei* phenotypes of interest as well as age, sex, and genetic principal components for 502,616 participants recruited into the UK Biobank dataset. Then for each phenotype, we excluded those with missing covariates or phenotypes. Due to incomplete access to individual level hypothyroidism in the UK Biobank, our analysis incorporates only 11 of the 12 traits reported in (Forgetta et al., 2022).

For calculating PRS scores, GWAS results for the 11 traits were taken from the Polygenic Score (PGS) catalog (https://www.pgscatalog.org/), and the numbers of SNPs in the PRS for different phenotypes are shown in Table 5). Only SNPs with MAF >0.01 were used when matching to the UKbiobank imputed genotypes (previously imputed with the IMPUTE4 program, https://jmarchini.org/software/, using the merged UK10K and 1000 Genomes phase 3 reference panels). The individual level phenotype data for each one of the 11 traits was collected from the UK Biobank browser (https://biobank.ndph.ox.ac.uk/).

### 2.3 Wisconsin longitudinal study

The Wisconsin Longitudinal Study (https://www.ssc.wisc.edu/wlsresearch/) (Herd et al., 2014) is a population cohort of over 10,000 individuals who graduated from high school in Wisconsin in 1957 and were followed intermittently until 2011. The data contain extensive information on lifestyle and behaviours as well as genetic data. We examine whether obesity, as measured by body mass index (BMI), influences Health-Related Quality of Life (HRQL), where the latter was measured by the Health Utility Index Mark-3. Unrelated individuals with BMI ≥25 were retained for analysis, resulting in a dataset containing 3023 subjects.

We focused on the imputed genetic data. Imputation was implemented using the software IMPUTE2 (Howie et al., 2009), which was based on a refined collection of genetic variants that passed quality control (Laurie et al., 2010), including filtering for minor allele frequency (MAF) ≥ 0.01, missing call rate < 2%, Hardy-Weinberg Equilibrium (HW) *p*-value $\geq 10^{-4}$ , etc. The performance of imputation was evaluated *via* BEAGLE allelic *r* ^2^ (Li et al., 2010) and gave a total of 3,683,868 SNPs (BEAGLE allelic *r* ^2^ no less than 0.3) used in our analysis. Basic descriptive statistics regarding the genotype and phenotype data can be found in Table 2.

**TABLE 2:** Descriptive statistics for the Wisconsin Longitudinal Study. *HRQL*: Health related quality of life was measured by the Health Utility Index Mark 3. BMI: Body Mass Index. s.d.: Standard deviation.

Variable	Metric	Value
Gender	Percentage	51
BMI	Mean (s.d.)	30.6 (4.93)
Age	Mean (s.d.)	71.2 (0.9)
Year of Education	Mean (s.d.)	13.8 (2.38)
HRQL	Mean (s.d.)	0.786 (0.227)
SNPs	Number available	3,683,868

## 3 Materials and methods

The standard, generic formula for a GRS is:

$$GRS_{i}=\underset{j\in S}{\sum }\beta_{j}g_{ij}$$ (1)

where *g*_*ij*_ are the genotypes for a set of individuals *i* = 1, … *N*, and for SNPs indexed by *j* ∈ **S**, where **S** is a selected set of SNPs. The same formula applies for PRS although set **S** will be larger. Often the genotypes will be coded as 0, 1, 2, counting the number of minor alleles at the SNP. In another common approach, the genotype data are centered and scaled to have mean zero and unit variance prior to score construction. How to choose the set **S** is a crucial question involving considerations of ancestry and linkage disequilibrium, as well as statistical significance thresholds and the analysis methodology used when associating the SNPs with a phenotype. The notation *β*_*j*_ represents the weight attributed to each copy of the minor allele; if genotypes are scaled then *β*_*j*_ must also be scaled correspondingly. The estimated coefficients ${\hat{\beta }}_{j}$ are frequently obtained from large, published GWAS for a particular phenotype; clearly the standard error of ${\hat{\beta }}_{j}$ will depend on the GWAS sample size and allele frequency at SNP *j* as well as methodology choices. Therefore, any inaccuracy or error in a genetic score could be due to having the wrong set of SNPs (**S**), the wrong coefficient estimates *β*_*j*_, or incorrect genotype measurements. Dudbridge (2013) and Chatterjee et al. (2013) have described how predictive accuracy (measured by *R* ^2^ for a continuous phenotype or by area under the curve (**AUC**) for a binary phenotype) depends on the heritability, sample size, and distributions of true effect sizes. AUC can be interpreted as the probability that a randomly selected case will have a higher score than a randomly selected control (Hanley and McNeil, 1982). A random classifier will yield an AUC of 0.5. Examining prediction accuracy for individuals, Ding et al. (2021) have estimated the width of an individual’s risk credible interval, and have shown that these widths vary with the magnitude of risk and with the genotype profile of the individual.

If we assume that all genotypes are accurate, and that a score contains the correct set **S**, variation in score performance across subgroups of the population or across different study contexts must be due to variability in the values of *β*_*j*_ across these subgroups. Such variability could either be due to different true values across subgroups, or to inaccurate estimated values. For example, a SNP in a gene that transcribes a sex-specific hormone could have different true effects in males versus females. Supplementary Materials SA, SB provide a simple algebraic look at bias and variance associated with one estimated SNP effect when it differs between two subgroups, and the consequences for a genetic score. Mean squared error may be smaller for subgroup-specific estimates, if there is a large enough difference between groups. On the other hand, particularly in small studies, imprecise and inaccurately-estimated coefficients are to be expected. Estimates ${\hat{\beta }}_{j}$ will have large standard errors and the most significant SNPs are likely to show estimates biased away from the null due to winner’s curse (Palmer and Pe’er, 2017). Replication of results, either through cross-validation or through use of an independent dataset, can provide insight into whether genetic effects have been over-estimated.

### 3.1 Meta-analysis, cross-validation and single nucleotide polymorphism selection in temporomandibular disorder data

When GWAS data are available from several separate datasets or cohorts, choices must be made for how to combine or aggregate information across the datasets to construct scores with the most accurate risk estimates. If there are differences between the true cohort-specific coefficients for some of the SNPs, with or without differences in disease prevalence, this aggregation choice will have a strong impact on the predictive performance of the cohort-combined score. In fact, if the cohorts are very different–e.g., different exposures or comorbidities–it may not be advantageous to combine.

When the score coefficients, *β*_*j*_, are estimated in the same data used to develop the genetic scores, estimates for the most significant SNPs will be biased away from the null (Palmer and Pe’er, 2017). To separate comparisons of modelling strategies from overfitting in our analyses of the TMD datasets, we implemented a careful data splitting strategy including training, validation and test datasets. First, all four cohorts were combined into one large dataset. Then we performed 5-fold cross-validation of all analyses described below, such that each training dataset contained 80% of the full dataset, and the validation and test datasets each contained 10% of the combined data. Sampling for the cross-validation was performed to ensure that balanced numbers of cases and controls were selected from each of the four TMD datasets at each split. Generally speaking, GWAS analyses were performed in the training datasets, parameter estimation in the validation sets, and estimation of performance in the test sets; specific details are provided below. All analyses were repeated over 10 random cross-validation splits of the data, and results are summarized by medians and interquartile ranges of performance metrics. Note that these resampling steps were designed so that the same datasets were used for each modelling strategy.

We implemented three modelling strategies:

• First, using the four-cohort combined training datasets (the C + T method), we tested association with TMD genome-wide using PLINK, employing a logistic regression for additive SNP effects, with age, sex, enrollment site and cohort as covariates and the first 10 principal components (**PCs**) of the genotype data. The PCs were calculated after merging the raw genotypes from all cohorts, to account for population stratification. The PRS was then calculated on a subset of genetic markers obtained after LD-clumping, which removes highly correlated SNPs, followed by P-value thresholding (see Results). We then used the validation set to re-estimate the coefficients of the covariates (age, sex, enrollment site, cohort and the top 10 PCs), as well as the coefficient of the calculated PRS, and then predicted TMD status on the test set.
• In contrast to the combined TMD data analysis, our second approach used meta-analysis to estimate single-SNP associations with weighted averages of cohort-specific SNP effects (Borenstein et al., 2007). Since all four studies contain both cases with TMD and controls, the meta-analytic estimates are:

$${\hat{\beta }}_{j}^{\mathrm{meta}}=\frac{\sum_{k=1}^{K}w_{jk}{\hat{\beta }}_{jk}}{\sum_{k=1}^{K}w_{jk}},$$ (2)

where the weights *w*_*jk*_ for study *k* = 1, … *K* are defined as *w*_*jk*_ = 1/*v*_*jk*_, and *v*_*jk*_ is the variance of ${\hat{\beta }}_{jk}$ for the *kth* study and for SNP *j*.

For this meta-analysis implementation, cohort-specific training datasets were used to obtain ${\hat{\beta }}_{jk}$ and their variance estimates, and the meta-analyzed summaries ${\hat{\beta }}_{j}$ . C + T was applied using the meta-analytic summary estimates for *p*-value thresholds, and the clumping was applied to data from all training data cohorts combined. The validation datasets were used to re-estimate the coefficients of the resulting PRS and the other covariates, and test data was used for estimation of the AUC.

• A third analysis of the TMD data used multivariable penalized logistic regression. When it is computationally possible, multivariable penalized models may more accurately clump or prune SNPs than any C + T method (Forgetta et al., 2020), while also more accurately estimating the non-redundant contribution of each retained SNP. Using the bigsnpr package in R (Privé et al., 2019), we fit penalized logistic regressions on the TMD training combined data. For this method, we did not use the validation set, because the bigsnpr package includes tuning parameter estimation while training the model. In addition to age, sex, enrollment site, cohort and the top 10 genetic principal components of ancestry, the penalized regression models included all SNPs with a combined GWAS p-value from the first modelling strategy (on the combined data) below *P*_*T*_, for a range of values for *P*_*T*_. The LASSO penalty was used to force some coefficients to be exactly zero, thereby performing simultaneous variable selection.

### 3.2 Single nucleotide polymorphism selection with the effector index, *Ei*

C + T depends on linkage disequilibrium patterns at each GWAS locus. However, algorithms based only on this population-level correlation structure do not take into account whether a SNP is likely to be functional or causal for the disease, or is merely correlated with such SNPs. Numerous methods have been developed to improve on C + T, such as PRS-CS (Ge et al., 2019), LDpred (Vilhjálmsson et al., 2015), LDpred-func (Márquez-Luna et al., 2021), LDAK (Speed et al., 2012), or SBayesR (Lloyd-Jones et al., 2019); most of these methods simultaneously consider the linkage disequilibrium and the association rather than doing so in 2 steps (as does C + T).

A complementary set of methods have been designed for identifying causal genes at a GWAS locus. These can be grouped into several classes: methods based expression Quantitative Trait Loci (eQTL) such as eCAVIAR (Hormozdiari et al., 2016) restrict attention to genes whose expression is influenced by associated SNPs; methods such as DEPICT (Pers et al., 2015) assume that functional annotation of genes will prioritize effectively; approaches such as MAGENTA (Segrè et al., 2010) leverage information in biological annotations; and methods that implement detailed fine-mapping (e.g., PAINTOR (Kichaev et al., 2014) use a combination of statistical arguments and functional annotations. In this latter category of methods, we recently published the Effector Index (*Ei*) for predicting which is the most likely causal gene at a locus showing multiple GWAS signals (Forgetta et al., 2022). For *Ei*, the predictions of the most likely causal gene are based on many types of information including features of the associated SNPs at a locus, and include the magnitudes of the $\hat{\beta }$ coefficients, linkage disequilibrium patterns, fine mapping results, and DNase hypersensitivity sites. The *Ei* algorithm was built on a dataset containing information for 12 quantitative traits and diseases for which highly-powered GWAS have been published, and for which several true causal genes are well known and validated (Forgetta et al., 2022). For each of the 12 traits, a set of putatively causal SNPs was selected and annotated at GWAS-identified loci. The *Ei* algorithm then used xgboost, a machine learning algorithm (Chen and Guestrin, 2016) to predict the most likely causal genes, based on carefully-constructed features derived from GWAS SNP summary statistics, SNP annotations, and locus characteristics.

We wished to ask whether gene prioritization methods could also be useful in SNP selection for PRS or GRS construction. This idea can be considered as similar to methods for improving SNP annotation. However, here we propose to estimate SNP contributions indirectly through their role in causal gene identification, rather than directly through annotation of the SNP genomic position. Using data from UK Biobank and by reverse engineering the *Ei* algorithm, we implemented a form of leave-one-out sensitivity analysis to investigate identify which SNPs had the largest influence on the causal gene predictions at each locus. In this exploration, we used the same dataset that was assembled to build the *Ei* algorithm (Forgetta et al., 2022). For each locus with a GWAS signal at any of the traits used to build *Ei*, we dropped each SNP one at a time from the *Ei* xgboost model, and calculated the changes in the predicted probabilities of causality for each gene. Then, we defined locus-specific weights by summarizing the changes at a locus across the locus-associated SNPs. If dropping a SNP results in a large change in the probability of a gene being causal, then we argue that it could be an important SNP, and we use this rationale to create locus-level weights. Define

$$\delta_{j,g}=\text{Ei}\left(g\right)-Ei{\left(g\right)}_{-j}$$ (3)

$$\delta_{j}=\frac{\sum_{g\in G_{j}}\delta_{j,g}}{\left|G_{j}\right|}$$ (4)

$$w_{j}=\frac{\left|\delta_{j}\right|}{\underset{j\in J}{\sum }|\delta_{j}|}$$ (5)

Where *Ei*(*g*) is the original *Ei* score for gene *g*, *Ei*(*g*)_−*j*_ is the updated *Ei* score for gene *g* obtained by re-estimation of the algorithm after removing SNP *j*. Therefore, *δ*_*j*,*g*_ captures the change in the predicted probability for gene *g* by removing SNP *j*. We use the notation *G*_*j*_ to denote the set of all genes related to SNP *j*, i.e. at the same locus. Then *δ*_*j*_ denotes the average difference of *δ*_*j*,*g*_ across the set of genes *G*_*j*_ for SNP *j*. Finally we defined *w*_*j*_ to be the weight assigned to SNP *j* in PRS construction, by summarizing the mean differences across all SNPs at the same locus (*J*) as SNP *j*.

### 3.3 Improving single nucleotide polymorphism selection in genetic risk scores for Mendelian randomization studies

In Mendelian randomization (MR), genetic data are used as instruments to infer whether a modifiable risk factor has a causal effect on a disease phenotype or trait of interest. A genetic variant must satisfy three core assumptions in order to be a valid instrument: (i) it is informative of the modifiable risk factor; 2) there is no association between the genetic variant and unmeasured confounders; 3) each genetic variant has no effect on the outcome of interest except through the modifiable risk factor of interest (e.g. Wang and Tchetgen Tchetgen, 2018).

Combining several genetic variants into GRS can enhance causal inference in observational studies (Pingault et al., 2018). Previously, selection of appropriate instruments has primarily relied on expert knowledge and detailed annotation of the genome. However, the recent explosion of validated and robust genetic associations through GWAS makes it tempting to consider a larger number of genetic variants as potential instrumental variables. Inclusion of more SNPs may enable creation of a stronger instrument that explains more of the variance in the risk factor, and hence enables a more accurate MR-derived causal estimate (Sleiman and Grant, 2010; Brion et al., 2013). However, some genetic variants included in GRS may not satisfy the MR assumptions (1)–(3). Therefore, for MR studies, construction of GRS and specifically SNP selection is subject to specific challenges.

One challenge is that the SNPs used to construct the GRS may violate assumption 3) due to pleiotropy, a phenomenon where a genetic variant may influence a disease or trait through independent pathways (Hemani et al., 2018). Inclusion of pleiotropic instruments in MR can lead to biased causal estimates (Burgess and Thompson, 2013). Therefore, new MR methods have been proposed for making valid causal inference in the presence of invalid instruments. One line of research has focused on one-sample study designs, and assumed that the number of valid instruments is subject to some minimal constraints. For example, Bowden et al. (2016) and Kang et al. (2016) provided consistent estimates by assuming that a majority of instruments (more than 50% of instruments) are valid. This assumption is known as the majority rule. More recently, Guo et al. (2018) and Windmeijer et al. (2019) considered a weaker assumption that the number of invalid instruments giving an equivalent Wald ratio is strictly less than the number of valid instruments; this assumption has been termed the plurality rule. For two-sample MR, when SNP-risk factor associations are determined in one dataset but risk factor-disease associations are measured in another, Zhao et al. (2020) used an adjusted profile score for constructing consistent causal estimates. Also, Ye et al. (2021) have proposed a debiased inverse-variance weighted (IVW) estimator.

A second challenge is that when the sample size is small to moderate (e.g. in the hundreds or thousands), GWAS will have limited power to detect small SNP effects. The industry-standard GWAS *p*-value threshold of 5 × 10^–8^ controls the family-wise error rate at 5%, but may exclude many SNPs with small effects. In other words, a genome-wide significance threshold applied to a small or moderate sample size study may result in few or even no SNPs for construction of a GRS. Therefore, it may be tempting to use a less stringent significance threshold to obtain a larger number of SNPs and hence a potentially-stronger GRS instrument. However, a less conservative threshold is likely to include more false positive associations, i.e. SNPs having spurious correlations with the risk factor of interest. We refer to such SNPs that are falsely selected as “spurious instruments”.

In Zhang et al. (2022), we showed that–as expected–including such spurious variables can bias causal effect estimates. We also showed that the spurious instruments behave similarly to each other. We then developed a resampling method that generates independent noise variables, and we used these resampled noise variables to help identify candidate instruments with spurious correlations with the risk factor. This strategy then allows us to disregard potentially spurious instruments, and hence to alleviate the effect of spurious instruments when constructing a GRS.

Using data from the Wisconsin Longitudinal Study, we compared performance of a standard GRS (Eq. 1) for BMI, to a GRS constructed with this resampling-based approach for excluding spurious instruments, i.e., a GRS from a smaller set of SNPs **S**. Both scores are then used to assess whether BMI is causally related to quality of life (HRQL) in the Wisconsin data. Unlike a standard GRS, this resampling method also uses information from the disease or outcome variable when performing SNP selection. Detailed methods and discussions can be found in Zhang et al. (2022).

### 3.4 Evaluation of genetic score performance

We evaluated each GRS’s or PRS’s ability to discriminate between cases and controls by determining the area under the receiver-operator characteristics curve (**AUC**). For continuous traits, we used the *R* ^2^ between predicted and observed values to assess performance. We also evaluated the number of SNPs retained in the GRS or PRS scores by different methods.

## 4 Results

### 4.1 Comparing single nucleotide polymorphism selection and polygenic risk scores performance in temporomandibular disorder

With data from the four TMD cohorts (Table 1), and implementing a repeated 5-fold cross-validation (see Methods), we compared the performance of three different methods for PRS construction: the Clumping + Thresholding (C + T) method applied to the combined dataset, the meta-analysis version of the C + T method (META C + T) and multivariable penalized logistic regression.

The best overall achieved prediction of TMD, measured by the median AUC across the 10 repetitions of the cross-validation, is obtained by the joint penalized PRS where all SNPs are included, as presented in Table 3. The predictive performances of the C + T and META-PRS models are comparable and do not vary much with the inclusion threshold *P*_*T*_, as opposed to the prediction performance of the joint model, which is highly affected by the value of *P*_*T*_. Indeed, for values of *P*_*T*_ ∈ {0.05, 10^–2^, 10^–3^, 10^–4^}, predictions for the joint PRS are less accurate than for the scores derived from univariable calculations. This is in line with previous findings which have shown that reprioritizing SNPs found by univariable tests reduces predictive power in penalized regression models (Abraham et al., 2012; Privé et al., 2019). This result can be explained by a bias-variance tradeoff. For values of *P*_*T*_ ∈ {10^–2^, 10^–3^, 10^–4^, 10^–5^}, almost all predictors that enter the model are selected, as can be seen in Table 4. Hence the low level of regularization encourages models with more predictors, which results in estimated coefficients that tend to overfit the training data. Therefore, these models will have lower bias, but higher variance, and they will not generalize adequately to new data. For values of *P*_*T*_ higher than 0.01, the number of predictors that enter the model increases drastically over the number of subjects in the sample, regularization is more important, and the predictive performance of the joint PRS increases. See also Supplementary Material SC.

**TABLE 3:** Median and interquartile range for AUC of different PRS models as a function of *p*-value threshold *P*_*T*_. For each model and value of *P*_*T*_, estimates are obtained by averaging results across ten repeated instances of 5-fold cross-validation.

*P*_*T*_	C + T PRS	META-PRS	Joint PRS
10^–5^	0.618 (0.0346)	0.612 (0.0383)	0.627 (0.0393)
10^–4^	0.615 (0.0401)	0.611 (0.0406)	0.586 (0.0345)
10^–3^	0.616 (0.0367)	0.614 (0.0375)	0.564 (0.0322)
10^–2^	0.612 (0.0323)	0.615 (0.0368)	0.591 (0.0348)
0.05	0.614 (0.0365)	0.615 (0.0335)	0.609 (0.0350)
0.1	0.616 (0.0333)	0.615 (0.0330)	0.621 (0.0295)
1	—	—	0.643 (0.0418)

**TABLE 4:** Median and interquartile range for the number of SNPs included in different PRS models, as a function of *p*-value threshold *P*_*T*_. For each model and value of *P*_*T*_, numbers are the average result across ten repeated instances of 5-fold cross-validation. For the Joint PRS, we present the number of predictors that were entered into the model, in addition to the number of predictors selected after LASSO regularization.

*P*_*T*_	C + T PRS	META-PRS	Joint PRS	
			Predictors in the model	Predictors selected
10^–5^	12 (4)	12 (3)	27 (3)	27 (3)
10^–4^	100 (11)	101 (12)	78 (7)	78 (7)
10^–3^	860 (46)	852 (46)	538 (33)	534 (29)
10^–2^	6530 (89)	6412 (77)	3848 (103)	3186 (72)
0.05	23 784 (134)	23 464 (202)	13 824 (143)	5920 (549)
0.1	39 916 (198)	39 496 (219)	22 994 (169)	4802 (855)
1	—	—	3 726 754 (21)	212 (497)

**FIGURE 1:**
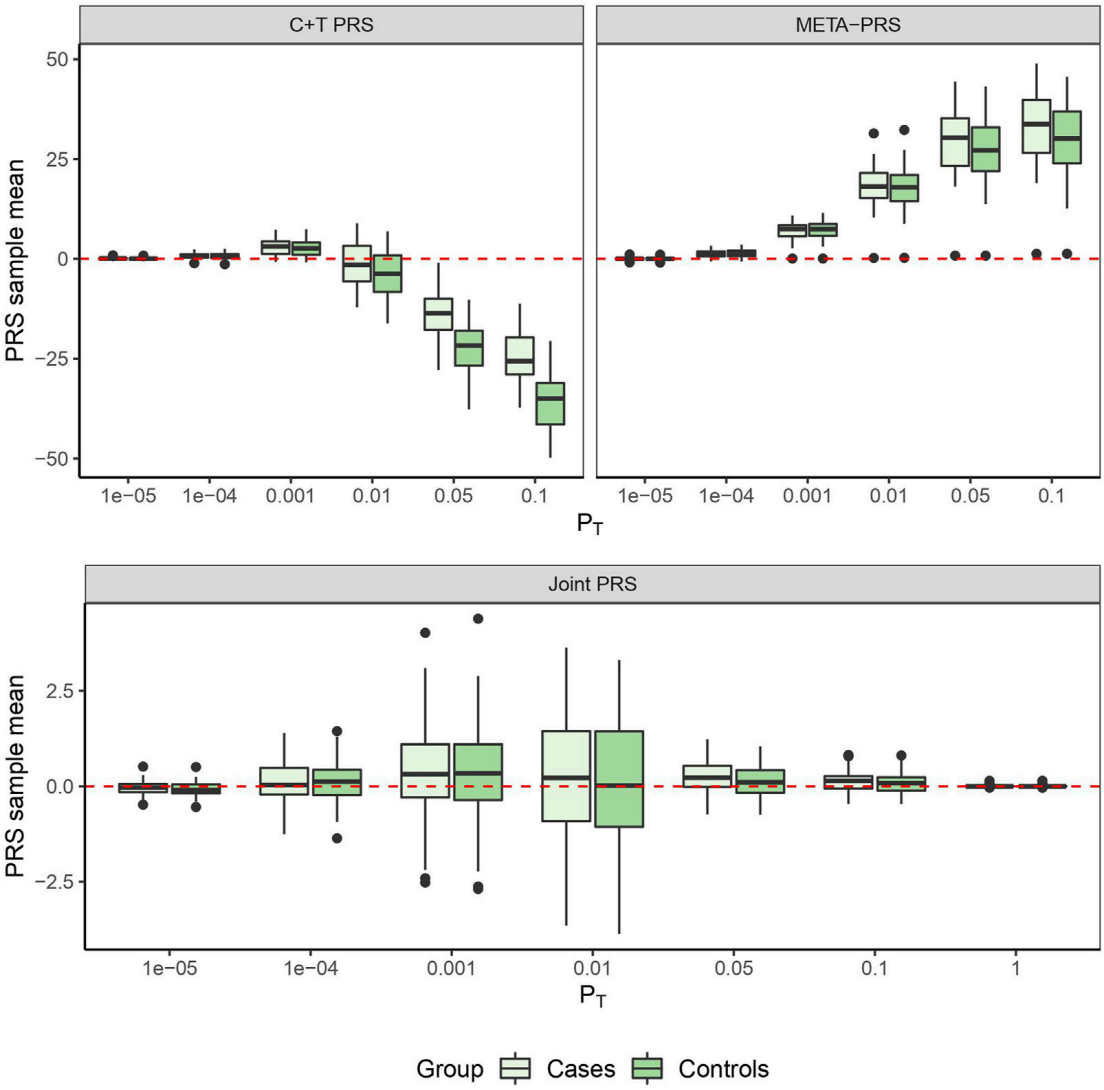
PRS sample means obtained from 10 times 5-fold cross-validation for different models as a function of *p*-value threshold *P*_*T*_.

In Figure 1, we compare for each method the distribution of the PRS cross-validation sample means for cases and controls separately, excluding the contribution from the non-genetic predictors. For the univariable C + T and META-PRS methods, as we increase the number of predictors in the model, distributions of sample means move away from zero, and variances of sample means increase. For the joint PRS, increasing the number of predictors in the model also increases variance of the PRS sample means until the number of predictors becomes significantly higher than the number of subjects. Thus, joint estimation potentially reduces prediction error by reducing bias in estimation of SNPs effects, while regularization, by simultaneously controlling the number of predictors retained in the model and reducing the size of estimates, avoids overfitting on the training data and reduces variance of predicted sample means in both cases and controls. Of note, even though the C + T PRS sample mean distributions discriminate well between cases and controls for higher values of *P*_*T*_, the model still performs poorly for predicting individual probabilities compared to the joint PRS, as assessed by the AUC values reported in Table 3.

### 4.2 Single nucleotide polymorphism prioritization through reverse engineering *Ei*

**FIGURE 2:**
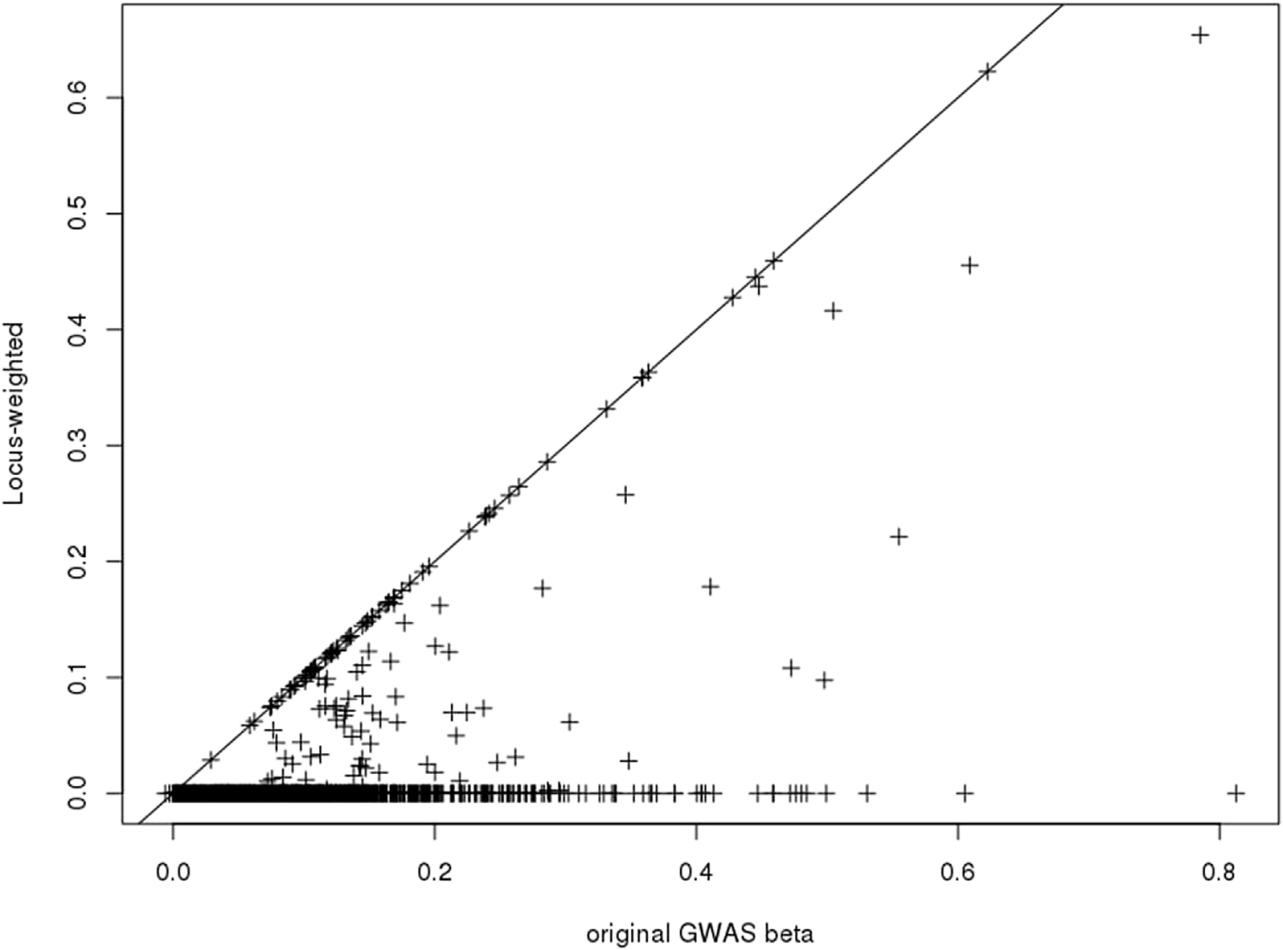
Scatter-plot showing the published GWAS association statistics ( $\hat{\beta }$ ; horizontal axis) against the *Ei*-weighted estimated *β* for diastolic blood pressure (vertical axis). Only SNPs which contributed to the construction of *Ei* are shown.

Only a subset of the SNPs included in the PGS catalog for any particular phenotype were used in the construction of the *Ei* algorithm, due to the annotation of associated SNPs (see Forgetta et al. (2022)). After re-estimating the *Ei* predictor leaving out each of these SNPs in turn, we used Eq. 3 to calculate the SNP specific changes for each related gene. Then for each SNP we summarized across genes to obtain its contribution score (Eq. 4). Finally, the contribution scores for SNPs at the same locus were summarized to create the locus-specific weights (Eq. 5). The resulting weights are illustrated in Figure 2 for one phenotype, diastolic blood pressure (**DBP**). For a subset of the SNPs, weights were exactly 1.0 (points on the diagonal line), and these are SNPs where there was only one potentially-causal gene at a GWAS locus. Most weights were smaller than 1.0, and many were zero. In fact, for DBP, 69 out of 139 (50%) GWAS significant SNPs in the *Ei* set were assigned locus-specific weights of 0; that is for DBP, the approach we proposed estimates 50% of these SNPs to have no influence on whether a nearby gene is causal.

We repeated a similar analysis for 10 additional UK Biobank phenotypes and then built PRS scores in three ways. Firstly, we calculated PRS scores using the published PGS catalog entries for these phenotypes (catalog entry IDs are in Table 5). Then we recalculated the PRS using only SNPs where we had obtained *Ei*-derived weights. On this subset, we calculated the PRS with and without using the *Ei* weights. The numbers of SNPs going into the published PRS, used in constructing the published *Ei* scores, and the numbers with non-zero weights following Eq. 5, are shown in Table 5 for each phenotype. Finally for each PRS method, we fit a regression model using the PRS as a covariate on a (randomly selected) 80% of the individuals, and predicted the results to a test set consisting of the rest of individuals with non-missing phenotype values. The prediction performances for each of the 11 phenotypes are measured with *R* ^2^ values (Table 5). For the *R* ^2^ (*Ei*_snp) and *R* ^2^ (*Ei*_weighted) columns of Table 5, SNPs without *Ei* weights were not included in the calculations.

**TABLE 5:** Predicted *R*-squared values, sample size and numbers of SNPs used, when using PRS to predict eleven phenotypes for three different PRS constructions. *Catalog ID*: the PGS catalog ID which was used to identify SNPs. *R* ^2^ *PGS*: *R* ^2^ for PRS scores using SNPs from the PGS catalog (https://www.pgscatalog.org/). *R* ^2^ *Ei*_snp: PRS scores built using only SNPs that were included in the *Ei* project. *R* ^2^ *Ei*_weighted: PRS scores built with (nonzero) SNP-specific weights as defined in the text. *Samples (N)*: Numbers of individuals included in training and test sets (combined) for calculation of *R* ^2^. *SNPs* (*PGS*): the number of SNPs in the corresponding PGS catalog that contributed to *R* ^2^ (*PGS*). *SNPs (Ei + PGS)*: the number of SNPs contributing to *R* ^2^ (*Ei*_snp), i.e., SNPs that were in the PGS catalog and used in the *Ei* project. *Ei + PGS+* *ω* ≠0: the number of SNPs contributing to *R* ^2^ (*Ei*_weighted). i.e., SNPs that were 1) in the PGS catalog; 2) used in the *Ei* project, and 3) had nonzero *Ei*-derived weights *ω*_*j*_.

Phenotype	Catalog (ID)	*R* ^2^ PGS	*R* ^2^ Ei_snp	*R* ^2^ Ei_weighted	Sample (N)	*SNPs* (*PGS*)	*SNPs* (*Ei + PGS*)	*SNPs* (*Ei + PGS+* *ω* ≠ *0*)
Calcium	PGS000676	0.028	0.011	0.010	425,150	12,239	85	39
Direct bilirubin (Dbilirubin)	PGS000681	0.249	0.049	0.049	394,374	3,067	36	16
Diastolic blood pressure (DBP)	PGS000302	0.049	0.031	0.031	394,374	961	139	70
EBMD (Estimated bone mineral density)	PGS000121	0.066	0.062	0.062	274,378	61	3	3
Glucose	PGS000684	0.021	0.015	0.013	15,593	3,279	27	13
Height	PGS000758	0.541	0.537	0.546	21,907	33,937	650	275
LDL (low-density lipoprotein)	PGS000824	0.072	0.022	0.018	463,556	808	437	165
RBC (red blood cell counts)	PGS000187	0.337	0.303	0.299	472,516	675	280	155
SBP Systolic blood pressure)	PGS000301	0.145	0.129	0.127	456,230	969	146	71
Type II Diabetes	PGS000330	0.021	0.017	0.015	486,866	6,437,379	482	157
Triglycerides	PGS000826	0.095	0.064	0.063	464,055	768	31	15

We used the recommended SNP selections from the PGS catalog when building the basic PRS, so the GWAS *p*-value thresholds and corresponding numbers of SNPs in each PRS vary across the phenotypes. Hence, the numbers of SNPs contributing to the causal gene predictions in the *Ei* algorithm can be very different from number of SNPs in the PGS catalog, since different selection strategies were used. Predictions using the PGS catalog have higher *R* ^2^ values for all traits except height, even though only about half of the phenotypes (calcium, Bilirubin, EBMD, glucose, height, and type II diabetes) used scores built from larger numbers of SNPs than went into the *Ei* algorithm. For example, for height, there are 33,937 SNPs in the PRS, and only 650 of them contribute to the *Ei* algorithm for this trait.

The performance of scores from the PGS catalog and scores from the *Ei* subset of SNPs are difficult to compare since SNP selection is so different. However, when comparing the *R* ^2^ columns that start from the *Ei* SNPs, incorporation of *Ei*-derived weights leads to very similar or slightly worse *R* ^2^ values for most phenotypes with the exception of height. Therefore, our *Ei*-weighting strategy, at least as currently implemented, does not reliably lead to improved PRS performance for these phenotypes. Of the 650 SNPs used in building the *Ei* predictor for height, only 275 had non-zero weights in our adaptation, and a similar ratio applies to many phenotypes. Despite this, the *R* ^2^ values changed very little, suggesting that the most important SNPs may be accurately highlighted.

### 4.3 Obesity and health-related quality of life in the Wisconsin longitudinal study

In our recent work (Zhang et al., 2022), we simulated an dataset with 500 samples and 50,000 candidate instruments (SNPs), in which only 9 candidates were truly related to the exposure, and all the others were noise variables. GWAS-like screening procedures were used to select the SNPs that appear relevant to the exposure, but with a liberal selection threshold. On average, 9 relevant instruments and 15 spurious instruments passed the screening steps.

We found that the valid instruments had effect estimates that were similar to each other, and also that the spurious instruments displayed similar estimates, although this latter group of estimates were very different from the true causal effect. As a result, the largest group of candidate instruments with similar causal effect estimates corresponded to the noise variables that have spurious correlations with the exposure! In fact, these spurious instruments had estimates that were close to the ordinary least squares (OLS) estimate, an estimate that one would expect to obtain without accounting for unmeasured confounding. In other words, the causal effect estimate from MR studies would be subject to a similar amount of confounding as OLS, if one blindly applies existing methods for causal inference with invalid instruments to construct the GRS.

Using the Wisconsin Longitudinal Study data, together with the resampling method developed in (Zhang et al., 2022), we estimated the effect of obesity (as measured by BMI) on Health-Related Quality of Life (HRQL). The original candidate set included 3,683,868 true genetic variants, we then generated the same number of pseudo SNPs or noise variables. By applying the proposed resampling procedure to the expanded set of 7,367,736 candidate SNPs in total, we estimated that there were only 3 valid instruments, which were used for the construction of GRS. We obtained a causal effect estimate of −0.039 (95% confidence interval [ − 0.052, −0.025]). For comparison, we also used a standard GRS that did not exclude spurious instruments (Guo et al., 2018). Starting from the true genetic variants only, there were 29 genetic variants selected for the GRS based only on the strength of the association, and the resulting causal effect was estimated to be −0.010 (95% CI [ − 0.015, −0.005]). Consistent with our findings from the simulations, the standard SNP selection-based GRS led to a causal effect estimate close to the OLS estimate −0.011 (95% CI [ − 0.013, −0.009]). We refer readers to Zhang et al. (2022) for detailed discussions.

For a simple comparison of this approach to robust methods, we ran an Egger regression on the 29 SNPs selected for the standard GRS approach. This led to a causal estimate of −0.010 with 95% CI [ − 0.012, −0.008], values that are similar to the OLS estimate, and still quite different from the one that eliminated spurious instruments. Hence, more accurately identifying the truly associated SNPs makes an important difference in the causal effect estimates.

## 5 Discussion

We have presented several case studies that show challenges and opportunities associated with with PRS or GRS construction and interpretation. The accuracy of SNP selection with C + T versus multivariable penalized models is examined through analysis of several datasets containing patients with TMD; and we show that in these data, SNP selection through multivariate penalized models rather than *p*-value based filters can be beneficial. The SNP selection strategy had a strong impact on PRS performance in new datasets. These results add to ongoing community discussion on marginal versus joint estimation, since penalized models only performed well when given a large set of SNPs to start. Initial pre-filtering can negatively impact their performance.

Also, using some data from UK Biobank, we touched on the potential benefits and risks associated with basing SNP selection through a new SNP annotation derived from an algorithm originally designed to predict causal genes at GWAS loci. One might expect that improved SNP selection for the GRS or PRS set **S** should lead to improved predictive accuracies. However, including estimated weights into the score construction may also increase the weighted score’s measurement error. For now, only a small subset of the SNPs in large published PRS scores were annotated by our algorithm, and this may explain why only small effects were seen.

We also illustrated, with an example, that improved identification of SNPs that are invalid instruments for Mendelian randomization substantially altered inference of the causal effect of BMI on HRQL. The potential confounding of PRS–phenotype (or GRS–phenotype) relationships is always a concern when the predictor (the PRS) is not easy to interpret or decode. When selecting valid instruments for MR, possibly due to certain study design biases, invalid SNPs may all be associated with the same unknown confounders, and this potential situation leads to a bias that is consistent across invalid SNPs. Unlike median-based or robust regression methods for MR, the resampling-based method used here identifies the invalid instruments directly so that they can easily be removed prior to score construction.

Differences in coefficients in subgroups of a population–sex-specific effects for example–can certainly be expected to decrease the predictive performance of PRS if these differences are not captured. However, the benefits associated with obtaining less biased estimates of $\hat{\beta }$ may be offset by the increased variance of the resulting estimate, a consequence of estimating the coefficient from a smaller sample size. We took one example of a well-studied disease just to see whether these kinds of subgroup differences might be of concern. We built three PRS for cardiovascular disease in UK Biobank data white British participants with and without type 2 diabetes (see Supplementary Material SD). Perhaps surprisingly, the PRS performed very similarly in the two subgroups when considered alone. However, in contrast, other covariates with known diabetes associations had very different impact in the two groups. Hence, differences in disease prevalence and in covariate profiles between datasets may have stronger influence on PRS performance than differences in the SNP effects.

We have discussed SNP selection strategies that consider the SNPs one at a time (i.e., a filtering approach), or jointly (i.e., a penalized or regularized model fitting procedure). When analyzing the TMD data, we found that pre-selection of SNPs through *p*-value filters influences the joint fitting performance. That is, filtering can lead to winner’s curse bias even after penalized model fitting.

It may be interesting to consider methods for constructing PRS that are designed to optimize for more than one criterion at the same time. That is, methods for pre-filtering or joint modelling could be combined with identification of valid, possibly causal variants (Swerdlow et al., 2016). Combining techniques may lead to further improvements in risk predictions.

## Data Availability

The summary GWAS data for the OPPERA, OPPERA2 and CPPC cohorts are available upon request from Dr. Luda Diatchenko (McGill University). The summary GWAS data for the SPB cohort are available upon request from Dr. Carolina Meloto (McGill University). Imputed data for each cohort for individual-level analyses are available upon request following granted access to dbGaP project phs000796.v1.p1. Data from the UK Biobank: https://www.ukbiobank.ac.uk/ were obtained through accession number 60755. Data from the Wisconsin study can be requested from: https://www.ssc.wisc.edu/wlsresearch.
