# Supplementary material for: Considering strategies for SNP selection in genetic and polygenic risk scores
Source: Front Genet. 2022 Oct 25;13:900595. doi: 10.3389/fgene.2022.900595 (PMC9930898; doi:10.3389/fgene.2022.900595)
Supplement: Supplementary file 1 [file DataSheet1.pdf]

SUPPLEMENTARY MATERIAL

A. Bias and variance

Suppose that β_j depends on an exposure or demographic variable X, such that we have $\beta_j(X)$; for simplicity, suppose that X is a binary variable with values 0 and 1. Define

$$\begin{aligned} E(\hat{\beta}_j(X_i)|X_i = 0) &= \beta_{j0} \\ E(\hat{\beta}_j(X_i)|X_i = 1) &= \beta_{j1}. \end{aligned}$$

For a single individual i, therefore,

$$\begin{aligned} Bias(PRS_i|X_i = k) &= E(PRS_i) - E(PRS_i|X_i = k) \\ &= \sum_{j\in\{S\}} g_{ij} E\left(\hat{\beta}_j - \tilde{\beta}_j(X_i = k)\right) \\ &= \sum_{j\in\{S\}} g_{ij}\left(p_0\beta_{j0} + p_1\beta_{j1} - \beta_{jk}\right), \end{aligned} \tag{1}$$

where expectation is taken with ($\tilde{\beta}$) or without ($\hat{\beta}$) considering the value of X_i. The notation p_0 represents the probability that $X_i = 0$, $p_1 = 1 - p_0 = P(X_i = 1)$, and β_{jk} is the subgroup specific true coefficient. The magnitude of the bias therefore depends on how different are β_{j0} and β_{j1}.

Similarly, define

$$\begin{aligned} Var(\hat{\beta}_j) &\approx \frac{(N_0 - 1)\sigma_{j0}^2 + (N_1 - 1)\sigma_{j1}^2}{(N_0 + N_1 - 2)(N)} \\ Var(\tilde{\beta}_j(X_i)|X_i = 0) &= \frac{\sigma_{j0}^2}{N_0} \\ Var(\tilde{\beta}_j(X_i)|X_i = 1) &= \frac{\sigma_{j1}^2}{N_1}, \end{aligned}$$

where N_0 and N_1 are the sample sizes in the subgroups. It is likely that σ_{j0}^2 and σ_{j1}^2 will be similar to each other; the differences between the two variances will likely be driven mostly by the sample sizes.

Depending on the magnitudes of the sample sizes in the subgroups and these 4 parameters, $(\beta_{j0}, \beta_{j1}, \sigma_{j0}, \sigma_{j1})$, the mean squared error of $\hat{\beta}_j$ will be minimized somewhere in the range $\{\tilde{\beta}_{jk}, \hat{\beta}_{jk}\}$. For a PRS summed over all SNPs, the estimate with the smallest mean squared error will also depend on how many of the SNPs have subgroup-specific parameters.

For example, suppose two equally sized subgroups exist in the data, each of size $N/2$. This implies that parameter estimation performed separately for each subgroup will lead to a estimates with variances approximately two times larger (standard errors that will be larger by roughly $\sqrt{(2)}$). Therefore, to benefit from the subgroup analyses, we could argue that the subgroup-specific estimates must differ sufficiently that bias^2 is reduced two-fold.

Supplement B shows formula for $Var(PRS_i)$ at the population level, which depends on the distribution of the subgroup-specific coefficients and standard errors, across all SNPs included in the PRS. We note

that any subgroup coefficient patterns will not be the same across all j, making predicting the benefit of subgroup analyses challenging.

B. Variance of PRS considering coefficients β_j as measured with error

Suppose that β_j depends on an exposure or demographic variable X, such that we have $\beta_j(X)$; for simplicity, suppose that X is a binary variable with values 0 and 1. We assume that the genotypes g_{ij} are known and not random.

Define

$$\begin{aligned} E(\hat{\beta}_j(X_i)|X_i = 0) &= \beta_{j0} \\ E(\hat{\beta}_j(X_i)|X_i = 1) &= \beta_{j1}. \end{aligned}$$

For a single individual i, therefore,

$$\begin{aligned} Bias(PRS_i|X_i = k) &= E(PRS_i) - E(PRS_i|X_i = k) \\ &= \sum_{j\in\{S\}} g_{ij} E\left(\hat{\beta}_j - \tilde{\beta}_j(X_i = k)\right) \\ &= \sum_{j\in\{S\}} g_{ij} \left(p_0\beta_{j0} + p_1\beta_{j1} - \beta_{jk}\right), \end{aligned} \tag{2}$$

where expectation is taken with ($\tilde{\beta}$) or without ($\hat{\beta}$) considering the value of X_i. The notation p_0 is the probability that $X_i = 0$, and $p_1 = 1 - p_0 = P(X_i = 1)$, and β_{jk} is the subgroup specific coefficient.

The variance can be written as

$$Var(PRS_i) = Var(\sum_{j\in\{S\}} \beta_j(X_i)g_{ij}).$$

Assume that there is independence of the genetic variants in the risk set $\{S\}$, achieved by use of appropriate pruning/clumping and thresholding, fine mapping, or haplotype construction. Hence, the variance can be written as

$$\begin{aligned} Var(PRS_i) &= \sum_j Var(\hat{\beta}_j(X_i)g_{ij}) \\ &= \sum_j g_{ij}^2 \left\{Var_x\left(E(\hat{\beta}_j(X_i)|X_i = x)\right) + E_x\left(Var(\hat{\beta}_j(X_i)|X_i = x)\right).\right\} \end{aligned} \tag{3}$$

The first term contains

$$\begin{aligned} E(\hat{\beta}_j(X_i)|X_i = x) &= \beta_{j0} \text{ if } X = 0 \\ &= \beta_{j1} \text{ if } X = 1, \end{aligned}$$

and therefore

$$Var_x\left(E(\hat{\beta}_j(X_i)|X_i = x)\right) = \beta_{j0}^2 p_0 + \beta_{j1}^2 p_1 - (\beta_{j0}p_0 + \beta_{j1}p_1)^2 \tag{4}$$

where $p_0 = P(X_i = 0)$ and $p_1 = 1 - p_0 = P(X_i = 1)$. In the second term, we assume

$$\begin{aligned} Var(\hat{\beta}_j(X_i)|X_i = x) &= \sigma_0^2 \text{ if } x = 0 \\ &= \sigma_1^2 \text{ if } x = 1. \end{aligned}$$

where these variances will depend very strongly on the sample sizes in the subgroups defined by X. Therefore

$$E_x\left(Var(\hat{\beta}_j(X_i)|X_i = x)\right) = \left(\sigma_{j0}^2 p_0 + \sigma_{j1}^2 p_1\right) \tag{5}$$

Finally,

$$Var(PRS_i) = \sum_{j \in \{S\}} g_{ij}^2 \left\{\beta_{j0}^2 p_0 + \beta_{j1}^2 p_1 - (\beta_{j0} p_0 + \beta_{j1} p_1)^2 + \left(\sigma_{j0}^2 p_0 + \sigma_{j1}^2 p_1\right)\right\} \tag{6}$$

C. Omitting important predictors may increase prediction error

In the standard C+T approach, we retain only the predictor with the most significant association with the trait of interest among a region of correlated markers. Let y_i be any continuous trait and assume the true causal model is given

$$y_i = X_i\beta + Z_i^T\gamma + \epsilon_i,$$

where $i = 1, ..., n$, X_i is a single genetic predictor, Z_i is a vector of p genetic predictors in the same region as X, $\epsilon_i \overset{iid}{\sim} \mathcal{N}(0, \sigma^2)$ and ϵ_i is independent of X_i and Z_i. Without loss of generality, we assume there is no intercept. If we ignore Z_i from the model, the OLS estimator for β is given by

$$\hat{\beta} = (X^TX)^{-1}X^Ty.$$

Assuming the predictor X_i is the most significant marker from the region, the mean prediction error of the PRS for the ith subject is equal to

$$\begin{aligned} E[\hat{y}_i - y_i \mid X, Z] &= E[X_i\hat{\beta} \mid X, Z] - X_i\beta - Z_i^T\gamma \\ &= X_iE\left[(X^TX)^{-1}X^Ty \mid X, Z\right] - X_i\beta - Z_i^T\gamma \\ &= \left(X_i(X^TX)^{-1}X^TZ - Z_i^T\right)\gamma \end{aligned} \tag{7}$$

From (7) we have that the mean prediction error of the PRS is proportional to the strength of the correlation between X and Z and the size of the omitted predictors effects $\gamma = (\gamma_1, ..., \gamma_p)$.

D. PRS FOR CARDIOVASCULAR DISEASE IN UK BIOBANK, SEPARATELY BY DIABETES

Using resources from the UK Biobank (Bycroft et al., 2018), we calculated three PRSs for coronary artery disease and evaluated their performance in predicting incident cardiovascular events amongst individuals with and without type 2 diabetes, separately. These three PRSs were developed using different methods: (1) Khera et al. (2018) developed a genome-wide PRS using LDPred (Vilhjálmsson et al., 2015) based on large-scale meta-analysis of GWASs; (2) Inouye et al. (2018) developed a “meta-GRS” by meta-analyzing

three genetic risk scores for cardiometabolic traits; and (3) Ye et al. (2021) developed a genomic functional annotation-informed PRS using AnnoPred (Hu et al., 2017)

Cardiovascular events were identified using a combination of self-reported medical history, hospital admission records, and surgical records of coronary revascularization (Inouye et al., 2018). Cardiovascular events that occurred after baseline assessment were considered incident cases. Those who had developed coronary artery disease prior to baseline assessment were not included in this analysis because the predictive performance of PRSs is known to attenuate substantially for these individuals (Howe et al., 2020). Discriminative power of each PRS in identifying individuals at an elevated level of cardiovascular risk was assessed using the C-index based on Cox proportional-hazards models. In addition, we examined and compared the predictive performance of other environmental and lifestyle risk factors conditioned on type 2 diabetes in two ways: (1) We tested the predictive performance of a multivariate model including a PRS, age, sex, genotyping array, recruitment centre, and the first 10 genetic principal components; and (2) we tested the predictive performance of two empirical risk predictors adopted in clinics, Pooled Cohort Equations (PCE) (Goff et al., 2014) and QRISK3 (Hippisley-Cox et al., 2017), which aggregated multiple clinical cardiovascular risk factors.

In the UK Biobank, genotyping data and complete cardiovascular risk factor information were available for 322,230 participants of White British ancestry without prevalent coronary artery disease. Our analysis thus focused on these individuals. We found that there was very little difference in the PRS' discriminative power between individuals with and without type 2 diabetes (Table S1, first 3 columns). Specifically, all of the three PRSs achieved a C-index of 0.61 for identifying incident coronary artery disease cases amongst in type 2 diabetes-free individuals, while the C-index decreased slightly to 0.60 for those with type 2 diabetes.

However, other environmental and lifestyle risk factors likely contribute very differently to the predictive performance (Table S1, columns 4 -8). As expected, joint predictor sets combining PRS and the other covariates mentioned above had consistently improved predictive performance compared to using PRSs alone, yet this improvement was more prominent amongst type 2 diabetes-free individuals (C-index = 0.76) than those having type 2 diabetes (C-index = 0.68). Results based on empirical predictors calculated from clinical risk factors also confirmed this discrepancy, with notably higher C-indices amongst type 2 diabetes-free individuals (PCE: 0.76 without type 2 diabetes vs. 0.65 with type 2 diabetes; QRISK3: 0.79 without type 2 diabetes vs. 0.69 with type 2 diabetes).

	Khera	Inouye	Ye	Khera + covar.	Inouye + covar.	Ye +covar.	PCE	QRISK3
Overall	0.610	0.614	0.613	0.757	0.758	0.758	0.764	0.795
non-T2D	0.610	0.611	0.613	0.759	0.759	0.760	0.755	0.788
T2D	0.597	0.601	0.597	0.678	0.681	0.679	0.647	0.693

Table S1. C-indices of PRSs and covariate-adjusted models in predicting incident coronary artery disease cases in the UK Biobank. T2D: type 2 diabetes. "+ covar." implies covariates (age, sex, genotyping array, recruitment centre, and first 10 genetic principal components) were included in models predicting incident coronary artery disease. Khera: PRS from Khera et al. (2018). Inouye: PRS from Inouye et al. (2018). Ye: PRS from Ye et al. (2021). PCE: Pooled cohort equations (Goff et al., 2014). QRISK3: from Hippisley-Cox et al. (2017)

REFERENCES

Bycroft, C., Freeman, C., Petkova, D., Band, G., Elliott, L. T., Sharp, K., et al. (2018). The uk biobank resource with deep phenotyping and genomic data. *Nature* 562, 203–209

Goff, D. C., Lloyd-Jones, D. M., Bennett, G., Coady, S., D'agostino, R. B., Gibbons, R., et al. (2014). 2013 acc/aha guideline on the assessment of cardiovascular risk: a report of the american college of cardiology/american heart association task force on practice guidelines. *Journal of the American College of Cardiology* 63, 2935–2959

Hippisley-Cox, J., Coupland, C., and Brindle, P. (2017). Development and validation of qrisk3 risk prediction algorithms to estimate future risk of cardiovascular disease: prospective cohort study. *bmj* 357

Howe, L. J., Dudbridge, F., Schmidt, A. F., Finan, C., Denaxas, S., Asselbergs, F. W., et al. (2020). Polygenic risk scores for coronary artery disease and subsequent event risk amongst established cases. *Human molecular genetics* 29, 1388–1395

Hu, Y., Lu, Q., Powles, R., Yao, X., Yang, C., Fang, F., et al. (2017). Leveraging functional annotations in genetic risk prediction for human complex diseases. *PLoS computational biology* 13, e1005589

Inouye, M., Abraham, G., Nelson, C. P., Wood, A. M., Sweeting, M. J., Dudbridge, F., et al. (2018). Genomic risk prediction of coronary artery disease in 480,000 adults: implications for primary prevention. *Journal of the American College of Cardiology* 72, 1883–1893

Khera, A. V., Chaffin, M., Aragam, K. G., Haas, M. E., Roselli, C., Choi, S. H., et al. (2018). Genome-wide polygenic scores for common diseases identify individuals with risk equivalent to monogenic mutations. *Nature genetics* 50, 1219–1224

Vilhjálmsson, B. J., Yang, J., Finucane, H. K., Gusev, A., Lindström, S., Ripke, S., et al. (2015). Modeling linkage disequilibrium increases accuracy of polygenic risk scores. *The american journal of human genetics* 97, 576–592

Ye, Y., Chen, X., Han, J., Jiang, W., Natarajan, P., and Zhao, H. (2021). Interactions between enhanced polygenic risk scores and lifestyle for cardiovascular disease, diabetes, and lipid levels. *Circulation: Genomic and Precision Medicine* 14, e003128
